# Evaluation of Retinal Nerve Fiber Layer and Macular Ganglion Cell Layer Thickness in Relation to Optic Disc Size

**DOI:** 10.3390/jcm12072471

**Published:** 2023-03-24

**Authors:** Jens Julian Storp, Nils Hendrik Storp, Moritz Fabian Danzer, Nicole Eter, Julia Biermann

**Affiliations:** 1 Department of Ophthalmology, University of Muenster Medical Center, 48149 Muenster, Germany; 2 Institute of Biostatistics and Clinical Research, University of Muenster, 48149 Muenster, Germany

**Keywords:** OCT, BMO, optic disc size, macrodisc, microdisc, macular ganglion cell layer, retinal nerve fiber layer, RNFL, thickness

## Abstract

To investigate whether optic nerve ganglion cell amount is dependent on optic disc size, this trial analyzes the correlation between Bruch’s membrane opening area (BMOA) and retinal nerve fiber layer (RNFL) thickness as well as macular ganglion cell layer thickness (mGCLT). Additionally, differences in RNFL and mGCLT regarding various optic disc cohorts are evaluated. This retrospective, monocentric study included 501 healthy eyes of 287 patients from the University Hospital Münster, Germany, who received macular and optic disc optical coherence tomography (OCT) scans. Rank correlation coefficients for clustered data were calculated to investigate the relationship between BMOA and thickness values of respective retinal layers. Furthermore, these values were compared between different optic disc groups based on BMOA. Statistical analysis did not reveal a significant correlation between BMOA and RNFL thickness, nor between BMOA and mGCLT. However, groupwise analysis showed global RNFL to be significantly decreased in small and large discs in comparison to medium discs. This was not observed for global mGCLT. This study extends existing normative data for mGCLT taking optic disc size into account. While the ganglion cell amount represented by the RNFL and mGCLT seemed independent of BMOA, mGCLT was superior to global RNFL in displaying optic nerve integrity in very small and very large optic discs.

## 1. Introduction

Optical coherence tomography (OCT) allows for the non-invasive, quantitative assessment of individual retinal layers, such as the retinal nerve fiber layer (RNFL) around the optic disc and macular ganglion cell layer thickness (mGCLT). OCT measurement results can be compared to a normative reference database and can, therefore, allow for the differentiation between pathological and physiological findings [1,2,3]. In clinical routine, RNFL values are consulted most often to complement fundoscopic findings of conspicuous optic disc morphologies. However, investigation of the mGCLT has proven to provide important information in addition to RNFL measurements [4]. mGCLT has been shown to be of high diagnostic value in optic neuropathies and optic disc abnormalities [5,6,7,8].

A quantitative dependence of retinal ganglion cells on optic disc area has been demonstrated for RNFL and for histological axon content [9] and may, therefore, also be postulated for mGCLT. This raises the question of whether mGCLT and RNFL correlate with optic disc size in OCT, and whether optic disc morphology should be considered when interpreting results of retinal layer thickness measurements.

This field of research remains controversial. While several studies report a positive correlation between RNFL thickness and optic disc area [10,11], others contrarily describe no significant association between RNFL thickness and optic disc area [12,13].

In clinical practice, optic discs will be described as small, medium or large, as this division can help in identifying certain risk factors associated with optic nerve head morphology, such as an increased risk of anterior ischemic optic neuropathy in small optic discs [14], or to differentiate pseudopapilledema from optic disc swelling in microdiscs. In turn, it can be challenging to discriminate macrodiscs from glaucomatous optic neuropathies due to an enlarged cupping. Traditionally, this attribution of optic discs to one group has been based on fundoscopic assessment or on measurement results of confocal scanning laser tomography (CSLT). In recent years, studies using OCT for the characterization of optic disc morphology have demonstrated that approaches based on Bruch´s membrane opening area (BMOA) can be used instead [7,15,16,17]; however, categorization and thresholds for micro- and macrodiscs are still not universally defined.

Since OCT is one of the most frequently used imaging modalities in ophthalmological practice, investigating the effect of optic disc size, more precisely BMOA, on retinal structures such as RNFL and mGCLT is of great interest. The primary aim of this work is to analyze RNFL and mGCLT in relation to optic disc size defined by BMOA in a healthy cohort. Secondly, RNFL and mGCLT findings will be compared between different study groups based on optic disc size. The diagnostic value of the findings reported will be evaluated. Furthermore, the results presented in this trial can act as a reference database for mGCLT and peripapillary RNFL thickness in normal and extreme optic disc size.

## 2. Materials and Methods

This monocentric, retrospective study included 501 eyes from 287 Caucasian patients, who were examined at the Department of Ophthalmology, Münster University Hospital, Germany between 1 January 2016 and 1 October 2022.

This study was approved by the ethics committee of the Medical Association of Westfalen-Lippe and the University of Münster (No.: 2022-493-f-S) and adhered to the tenets of the Declaration of Helsinki.

We conducted a search in the electronic patient file system FIDUS (Arztservice Wente GmbH, Darmstadt, Germany) filtering for patients who received both a macular and optic disc OCT (Spectralis^®^, Heidelberg Engineering GmbH, Heidelberg, Germany) in at least one eye. Patients were only eligible to be included in the study if both macula and optic disc OCT scans were conducted on the same day or at least in a time span of no more than 1 month. Only healthy eyes were included in the study, resulting from a holistic ophthalmological examination. Patients were not eligible to be included if any of the following exclusion criteria applied: higher myopic refraction errors (spherical equivalent of <−6.0 diopters), any retinal or optic nerve diseases or congenital anomalies. Furthermore, patients with central nervous system disorders or neurotoxic drug intake were excluded, except for patients taking quensyl with no signs of retinopathy on ERG. Artifacts and low quality in macular or optic disc scans were additional exclusion criteria.

OCT images were all taken in the same location under the same conditions by expert operators. Scans of the macula and optic disc were reviewed by an expert examiner (N.H.S.). Further, boundaries of the BMO were verified and adjusted if the automatic annotation software failed to properly place BMO boundary markers.

Data were recorded in the spreadsheet software Microsoft Office Excel (Microsoft, Redmond, WA, USA; Version 16.71). Descriptive data are presented as mean ± standard deviation (SD).

Global RNFL represents the mean value of all RNFL sectors and is provided automatically by the Heidelberg system. In accordance with this parameter, global mGCLT was defined as the mean value of all mGCLT sectors and was calculated separately.

**Figure 1:**
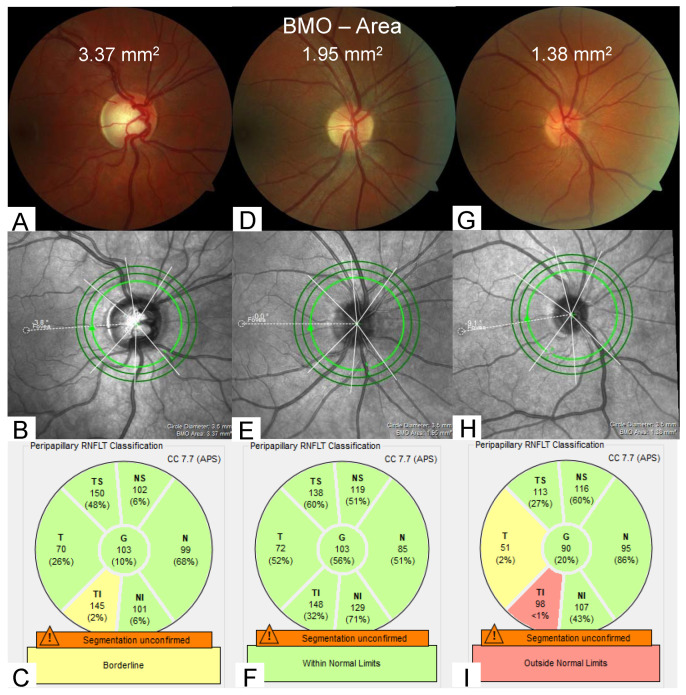
Examples for large, medium and small optic discs based on BMOA in fundoscopic view and optical coherence tomography scans. Upper row: fundus photographs of optic discs. Middle row: infrared images of the corresponding optic discs and illustration of the 3.5 mm, 4.1 mm and 4.7 mm diameter scan circles around the center of the optic nerve. Lower row: RNFL measurements of the corresponding optic discs (3.5 mm diameter) and valuation based on the database of Heidelberg Engineering GmbH. (**A**–**C**): large disc with a BMOA = 3.37 mm^2^, (**D**–**F**) medium disc with a BMOA = 1.95 mm^2^, (**G**–**I**): small disc with a BMOA = 1.38 mm^2^. (**C**,**F**,**I**): values for RNFL measurements. G = global RNFL; NS = nasal superior RNFL; N = nasal RFNL; NI = nasal inferior RNFL; TI = temporal inferior RNFL; T = temporal RNFL; TS = temporal superior RNFL.

The BMOA to RNFL correlation was firstly evaluated for global RNFL and subsequently for individual sectors of RNFL (Figure 1).

**Figure 2:**
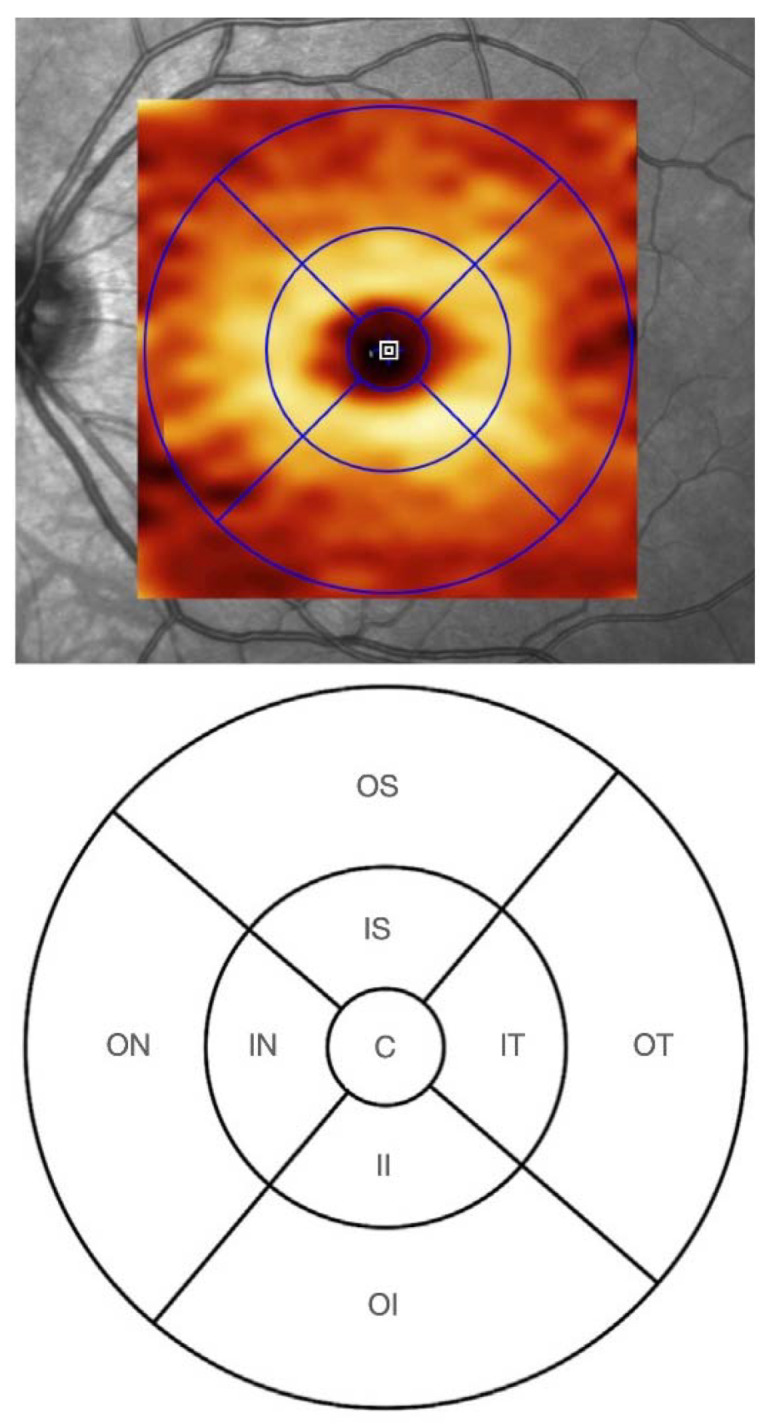
Location map for mGCLT data according to the Early Treatment Diabetic Retinopathy Study (ETDRS) grid. ETDRS sectors are labeled according to their location in relation to the fovea. C = Central area; IN = inner nasal; ON = outer nasal; II = inner inferior; OI = outer inferior; IT = inner temporal; OT = outer temporal; IS = inner superior; OS = outer superior.

Likewise, the correlation between mGCLT and BMOA was firstly tested for global mGCLT and secondly for the individual mGCLT sectors. The latter are based on the Early Treatment Diabetic Retinopathy Study (ETDRS) grid (Figure 2).

In a next step, eyes were assigned to respective optic disc groups according to their BMOA.

Due to the lack of accepted cut-off values in BMO measurements by OCT, the allocation to the three cohorts was done as an approximation based on the HRT (Heidelberg Retina Tomograph) definition: Group 1 “small discs” (*n* = 80; BMOA: <1.63 mm^2^), group 2 “medium discs” (*n* = 298; BMOA: 1.63–2.43 mm^2^), group 3 “large discs” (*n* = 126; BMOA: >2.43 mm^2^). Figure 1 displays examples for small, medium and large discs with their respective infrared images and RNFL values. In addition to this HRT-based division, groupwise comparison was also conducted via a quartile-based approach, comparing the 5% smallest and 5% largest optic discs to the remaining 90% (intermediate).

### Statistical Analysis

For each retinal layer thickness variable, we computed correlations with BMOA to analyze the dependence between the variables. Since the normal distribution assumption could not be ensured, rank correlations were computed. However, the standard Spearman correlation coefficient does not take the clustering structure of our data into account. Hence, correspondingly adapted methods from Rosner et al. [18] with data from patients of which both eyes are available (*n* = 214) were applied. For each correlation coefficient, we also report 95% confidence intervals. No adjustments for multiple testing were made here, as our analyses are of an exploratory nature.

In order to discover non-monotonic effects, we executed pairwise comparisons of all variables between the three groups of eyes based on the HRT definition, and also between the three groups obtained by grouping the smallest 5% of BMOA, the largest 5% of BMOA and the remaining mean BMOA of the sample together. In order to account for the dependency structure, we applied a corresponding rank sum test as suggested by Rosner et al. [19] and implemented by Jiang et al. [20]. In keeping with the exploratory nature of our analysis, we report Bonferroni-adjusted *p*-values separately for each variable, i.e., the *p*-values are multiplied by 3 for the three comparisons made for each variable (small vs. medium discs, small vs. large discs, medium vs. large discs). Additionally, we report median values of the layer thickness variables for each group.

We report 95% confidence intervals that do not contain zero and *p*-values falling below 0.05 as significant findings. However, the purpose of this study is purely exploratory, and these findings should, therefore, be treated with care or confirmed in a separate study.

Statistical analysis was performed using R, version 4.1.2 [21]. The package clusrank [20] was used to execute the rank sum tests from [19] and the package ggplot2 [22] was used to create plots.

## 3. Results

501 eyes from 287 patients were included in this trial. Study population characteristics are summarized in Table 1.

**Figure 3:**
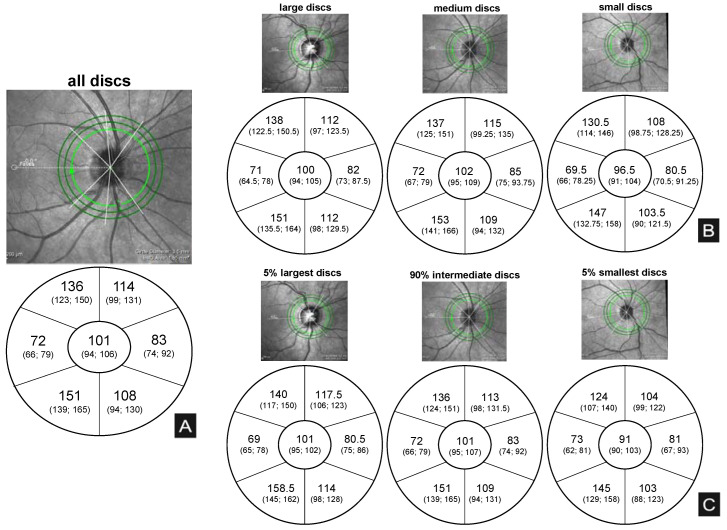
Illustration of median (25% quartile, 75% quartile) RNFL thickness values (µm). Note that, while right eye images are displayed as examples, the values shown were calculated on the basis of both right and left eye measurements. (**A**): median RNFL values for the entire patient population. (**B**): median RNFL values for the different cohorts based on HRT division. (**C**): median RNFL values for the different groups based on quartile division (5-90-5).

**Figure 4:**
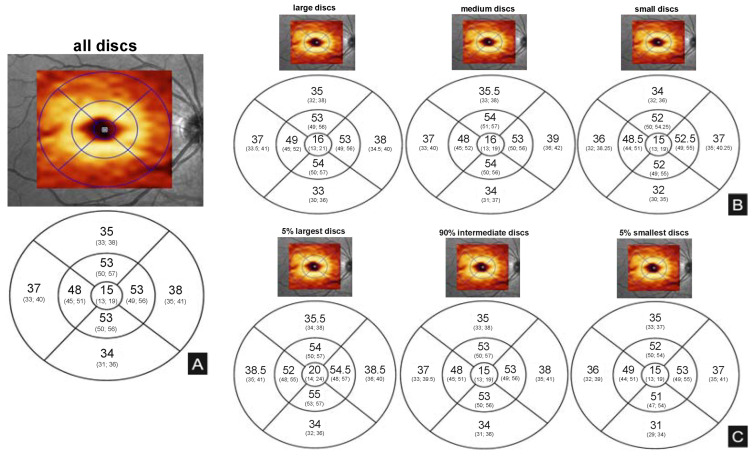
Illustration of median (25% quartile, 75% quartile) mGCLT values (µm). Note that, while right eye images are displayed as examples, the values shown were calculated on the basis of both right and left eye measurements. (**A**): median mGCLT values for the entire patient population according to the Early Treatment Diabetic Retinopathy Study (ETDRS) grid. (**B**): median mGCLT values for the different cohorts based on HRT division. (**C**): median mGCLT values for the different groups based on quartile division (5-90-5).

Nominal values for RNFL and mGCLT are summarized in Figure 3 and Figure 4.

**Figure 5:**
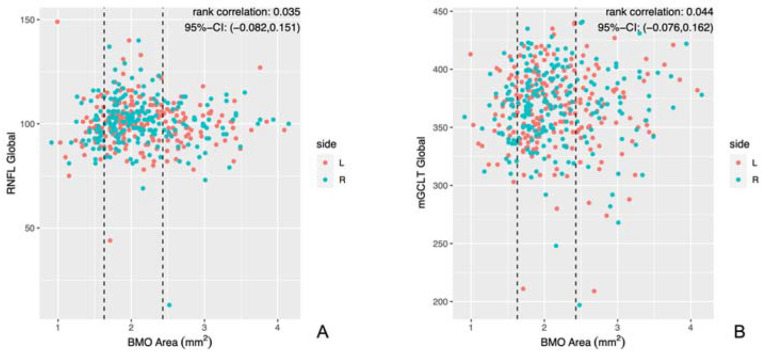
Scatter plots of global RNFL and global mGCLT in µm in relation to BMOA. (**A**): scatter plot of global RNFL and BMOA; dashed lines show bounds grouping by HRT definition. (**B**): scatter plot of global mGCLT and BMOA; dashed lines show bounds grouping by HRT definition. L = left eye; R = right eye.

Statistical analysis did not show any significant correlation between global RNFL and BMOA, nor between global mGCLT and BMOA (Table 2 and Figure 5).

### 3.1. Correlation Analysis

While a small effect could be seen for the central area of the mGCLT, no significant correlation could be demonstrated for the remaining individual sectors of the RNFL and mGCLT (Table 3 and Figure S1).

### 3.2. Optic Disc Groups (HRT Division)

Statistical analysis of the secondary hypothesis looked at possible differences between BMO-based optic disc size groups. In the HRT division, global RNFL differed significantly between small and medium discs as well as between medium and large discs. In contrast, global mGCLT did not show significant differences among the various cohorts (Table 4).

Results of the HRT-based comparison of the individual RNFL and mGCLT sectors among the three cohorts are displayed in Table S1. Statistical analysis revealed significant differences between small and medium discs in various RNFL and mGCLT sectors as well as significant differences between medium and large optic discs in the nasal sector of the RNFL.

### 3.3. Optic Disc Groups (Quantile Division)

To analyze the largest and smallest optic discs based on BMOA, this study also allocated the data based on a quantile division (5-90-5), comparing the 5% smallest optic discs, the 5% largest optic discs and the residual 90% intermediate discs. Results of this quantile-based approach are summarized in Table 5. Results of the quantile-based comparison of the individual RNFL and mGCLT sectors among the three groups are displayed in Table S2.

## 4. Discussion

The results of this retrospective study can be summarized as follows: Statistical analysis did not reveal a significant correlation between BMOA and RNFL thickness, nor between BMOA and mGCLT. Groupwise analysis showed global RNFL to be significantly decreased in micro- and macrodiscs when compared to medium sized discs. This was not observed for global mGCLT. This study extends existing normative data for mGCLT taking optic disc size into account.

This is the first study to examine the relationship between BMOA and RNFL, as well as between BMOA and mGCLT, using OCT in a large healthy study population.

In this monocentric analysis, neither RNFL nor mGCLT correlated significantly with optic disc size determined by BMOA.

However, noticeable differences were found among various optic disc groups, showing that while in the HRT division, global RNFL was reduced in small and large discs in comparison to medium discs, global mGCLT did not differ between these cohorts. In the quantile-based approach, this observation was reproducible for the smallest optic discs. These findings imply that mGCLT is affected less by optic disc size anomaly than RNFL thickness.

This observation is in line with data reported by Seo et al. [10]. The authors investigated the relation between optic disc size determined by CSLT and axial length on RNFL and ganglion cell–inner plexiform layer (GCIPL) in healthy individuals. While GCIPL and GCL are not entirely equivalent, both encompass similar retinal structures and can be considered equivalent with regard to their significance in clinical diagnostics [23]. While Seo et al. did not observe a significant correlation between GCIPL thickness and optic disc size, they report a positive correlation between optic disc size and RNFL thickness [10]. This is in line with reports by Savini et al., who describe a positive correlation between RNFL and optic disc size determined by OCT in their study of 54 healthy eyes [11]. Interestingly, we did not observe a noticeable correlation between RNFL or mGCLT and optic disc size in our study population. It should, however, be noted that optic disc size determination in the study by Savini et al. was based on the identification of the retinal pigment epithelium/choriocapillaris border and the addition of a 150 µm margin in papillary OCT scans, rather than BMO, which limits comparability. For the same reason, comparability to the CSLT based approach by Seo et al. is also limited. Nevertheless, the findings reported by Seo et al., and the findings reported in our study, consistently hint toward relatively constant values of the GCL/GCIPL irrespective of optic disc size variability [10].

While influencing factors on RNFL measurements are well described in the literature [24,25,26,27,28,29,30,31], there are little data on parameters that affect measurements of the mGCLT, such as axial length and spherical equivalent [10,32,33]. The findings of this study suggest that the size of the optic disc has little to no influence on mGCLT measurement results.

Optic disc size is a key aspect for correct optic disc assessment. Defining universally acknowledged thresholds to discriminate small from medium and large optic discs has posed a challenge due to the large variety of available methods [34,35,36]. Historically, optic disc size classification has been conducted during slit-lamp examination or on the basis of fundus photographs, both of which only allow for a certain degree of precision. For a long time, the assessment of the exact morphology and size of the optic disc was limited to findings in histological examinations [9]. The advances in retinal imaging of the past decades have enabled quantitative and reproducible measurements of optic disc parameters. CSLT devices have been applied extensively in order to investigate optic disc morphology. Nowadays, with the advancement of OCT, optic disc morphology can be computed three-dimensionally, allowing for precise illustration of optic nerve anatomy. Most importantly, OCT is capable of precisely identifying the termination of Bruch’s membrane, the anatomical landmark determining optic disc size, making BMOA a suitable anatomical structure for the assessment of optic nerve head morphology and disc size [37]. When assessing BMOA, it is important to rule out possible imaging artifacts that might otherwise confound tomographic scanning results, such as signal voids in Bruch’s membrane caused by overlying vessels.

Reports on the exact determination of optic disc size based on HRT and OCT are inconclusive. While some authors report no correlation between HRT and OCT optic disc measurements [38], Cazana et al. have recently demonstrated transferability of HRT measurements to an OCT-based BMOA assessment. They report a BMOA of ≥2.19 mm^2^ to resemble the adequate threshold value for optic discs to be considered macrodiscs; however, they did not include microdiscs in their analysis [15]. Therefore, as definitive BMOA reference values for micro-, norm- and macrodiscs remain elusive, this study adhered to the traditional HRT division for optic discs size classification and added a 5% quantile approach in order to examine extreme optic nerve heads.

Our data on the average GCL thickness are in line with normative data reported by a number of spectral-domain OCT trials [32,39,40,41]. Similar to our study, Invernizzi et al. investigated retinal layer thickness in 200 Caucasian patients [32]. Studies that investigated patients of Asian descent [40,41,42] also report absolute values for mGCLT, which are comparable to the median values presented here. While some of these studies have analyzed correlations between systemic parameters, such as gender and age, and thickness of different retinal layers, they do not report on the correlation between retinal layer thickness and optic disc size.

This study used the Heidelberg Spectralis^®^ Spectral-domain OCT (Heidelberg Engineering GmbH, Heidelberg, Germany), which comes with an integrated, color-coded reference database to help classify individual optic disc measurements. This reference database consists of 246 eyes of 246 patients, of whom 61 had a BMOA < 1.50 mm^2^ and 8 had a BMOA > 2.50 mm^2^. The authors of this manuscript are under the impression that micro- and macrodiscs can pose a challenge for the system when it comes to accurately rating RNFL values. Healthy individuals with very large or very small optic discs are frequently referred to our clinic due to abnormalities in the color-coded RNFL measurements (as demonstrated in Figure 1). Further work-up usually rules out any form of underlying disease which could cause such changes. We assume, therefore, that rather than being of actual pathological relevance, these RNFL abnormalities might occur due to a limited number of cases with optic disc anomalies being included in the reference database. This study included patients with normal visual function. Since our approach revealed mGCLT to be more stable in regards to changes in optic disc size, we suggest consulting mGCLT in micro- or macrodiscs in addition to RNFL measures if RNFL values appear implausible.

### Limitations

As has been shown by several authors, axial length/spherical equivalent can exert a significant effect on RNFL, mGCLT and BMO measurements [10,28,29,30,32,43]. By excluding patients with a spherical equivalent <−6 diopters, we limited a possible influence on the results in this study. Cognitive function, vascular health, age, ethnicity, OCT signal strength and gender have further been identified as confounding parameters for both RNFL and mGCLT measurements [24,25,26,27,44,45,46]. Since these parameters differ among trials, comparison of results must be done with caution. For instance, as this trial only included patients of Caucasian descent, our findings may not be transferable to other study populations.

One major limitation of this study lies in its design. Due to its retrospective nature, we are unable to comment on prospective estimates related to optic disc size and OCT measurements. Further prospective studies are needed here.

This study included patients with normal visual function who passed a holistic ophthalmological examination to exclude eye diseases. However, in daily clinical practice, early or preperimetric changes in retinal ganglion cell number and integrity may happen to soma and axon at different timepoints; for example, optic nerve compression may alter RNFL thickness prior to mGCLT, and mitochondrial optic neuropathy might affect mGCLT prior to RNFL thinning. Thus, taking other diagnostic modalities and parameters into account is crucial for the correct interpretation of OCT values.

## 5. Conclusions

To summarize, we did not observe a noticeable correlation between RNFL or mGCLT and BMOA in this study. In contrast to RNFL, mGCLT appeared to be independent of optic disc size in this cohort of healthy patients, thus suggesting that mGCLT should be consulted when investigating large or small discs and inconclusive findings in OCT–RNFL analysis. The display of RNFL and mGCLT standard values for various disc size groups in this study can further help distinguish pathological from physiological findings in clinical practice.

## Figures and Tables

**Table 1:** General patient characteristics. Values are presented as absolute numbers (%) or as median (25% quartile; 75% quartile).

*n* (Eyes)	501 (100%)
*n* (patients)	287 (100%)
age (years)	35 (16; 56)
gender (M:F)	210 (42%):291 (58%)
*n* (eyes) according to optic disc size (HRT division):	
large (2.43–4.15 mm^2^)	123 (25%)
medium (1.63–2.42 mm^2^)	298 (59%)
small (0.91–1.62 mm^2^)	80 (16%)
*n* (eyes) according to optic disc size (quantile division):	
largest (3.30–4.15 mm^2^)	25 (5%)
intermediate (1.41–3.30 mm^2^)	451 (90%)
smallest (0.91–1.40 mm^2^)	25 (5%)
study eye (R:L)	249 (50%):252 (50%)
median visual acuity (logMAR)	0.10 (0.00; 0.20)
median spherical equivalent:	0.00 (−0.75; 0.63)
per group (HRT division)	
large	0.00 (−1.13; 0.63)
medium	0.00 (−0.75; 0.63)
small	0.00 (−0.38; 0.66)
per group (quantile division)	
large	−0.25 (−1.25; 0.13)
medium	0.00 (−0.63; 0.69)
small	0.00 (−1.50; 1.50)

*n* = number, M = male; F = female, R = right, L = left, logMAR = logarithm of minimum angle of resolution, HRT = Heidelberg Retina Tomograph.

**Table 2:** Estimates and confidence intervals for correlation analysis between global RNFL and BMOA, as well as for correlation analysis between global mGCLT and BMOA.

	*Estimate*	*Lower 95% CI*	*Upper 95% CI*
*RNFL global*	0.04	−0.08	0.15
*mGCLT global*	0.04	−0.08	0.16

CI = confidence interval.

**Table 3:** Estimates and 95% confidence intervals of rank correlation coefficients of the individual RNFL and mGCLT sectors with BMOA.

*RNFL*	*Estimate*	*Lower 95% CI Bound*	*Upper 95% CI Bound*
*NS*	−0.04	−0.15	0.08
*N*	0.02	−0.09	0.14
*NI*	0.05	−0.07	0.16
*TI*	0.06	−0.06	0.17
*T*	−0.02	−0.14	0.10
*TS*	0.09	−0.02	0.21
*mGCLT*			
*C*	0.11	0.00	0.23
*IN*	0.02	−0.09	0.14
*ON*	−0.07	−0.18	0.05
*II*	0.06	−0.05	0.18
*OI*	0.00	−0.12	0.11
*IT*	0.09	−0.03	0.20
*OT*	0.05	−0.07	0.16
*IS*	0.03	−0.08	0.15
*OS*	0.01	−0.11	0.13

CI = confidence interval. RNFL sectors: NS = nasal superior; N = nasal; NI = nasal inferior; TI = temporal inferior; T = temporal; TS = temporal superior; mGCLT sectors: C = central area; IN = inner nasal; ON = outer nasal; II = inner inferior; OI = outer inferior; IT = inner temporal; OT = outer temporal; IS = inner superior; OS = outer superior.

**Table 4:** Differences in global RNFL and global mGCLT between optic disc cohorts based on HRT division. *p*-values ≤0.05 are highlighted in bold. Median thickness values are given in µm.

	*Small vs. Medium*	*Medium vs. Large*	*Small vs. Large*	*Median Thickness Small*	*Median Thickness Medium*	*Median Thickness Large*
*RNFL Global*	***<0.01***	***0.05***	0.42	96.50	102.00	100.00
*mGCLT Global*	0.08	1.00	0.94	40.11	41.22	40.89

**Table 5:** Differences in global RNFL and global mGCLT between optic disc cohorts in the 5 percent quantile division. Variable-wise Bonferoni-corrected *p*-values ≤ 0.05 are highlighted in bold. Median thickness values for the respective groups are given in µm.

	*Smallest vs. Intermediate*	*Intermediate vs. Largest*	*Smallest vs. Largest*	*Median Thickness Smallest*	*Median Thickness Intermediate*	*Median Thickness Largest*
*RNFL Global*	***0.05***	1.00	0.37	91.00	101.00	101.00
*mGCLT Global*	0.83	0.25	0.23	37.00	38.00	38.50

## Data Availability

Not applicable.

## Supplementary Materials

The following supporting information can be downloaded at: https://www.mdpi.com/article/10.3390/jcm12072471/s1, Figure S1: Scatter plots of RNFL sectors in relation to BMOA; Table S1: Differences in individual RNFL and mGCLT sectors between optic disc cohorts based on HRT division; Table S2: Differences in individual RNFL and mGCLT sectors between optic disc cohorts based on quantile division.
