# Supplementary material for: Evaluation of Retinal Nerve Fiber Layer and Macular Ganglion Cell Layer Thickness in Relation to Optic Disc Size
Source: J Clin Med. 2023 Mar 24;12(7):2471. doi: 10.3390/jcm12072471 (PMC10095471; doi:10.3390/jcm12072471)
Supplement: Supplementary file 1 [file jcm-12-02471-s001.zip › Figure S1.pdf]

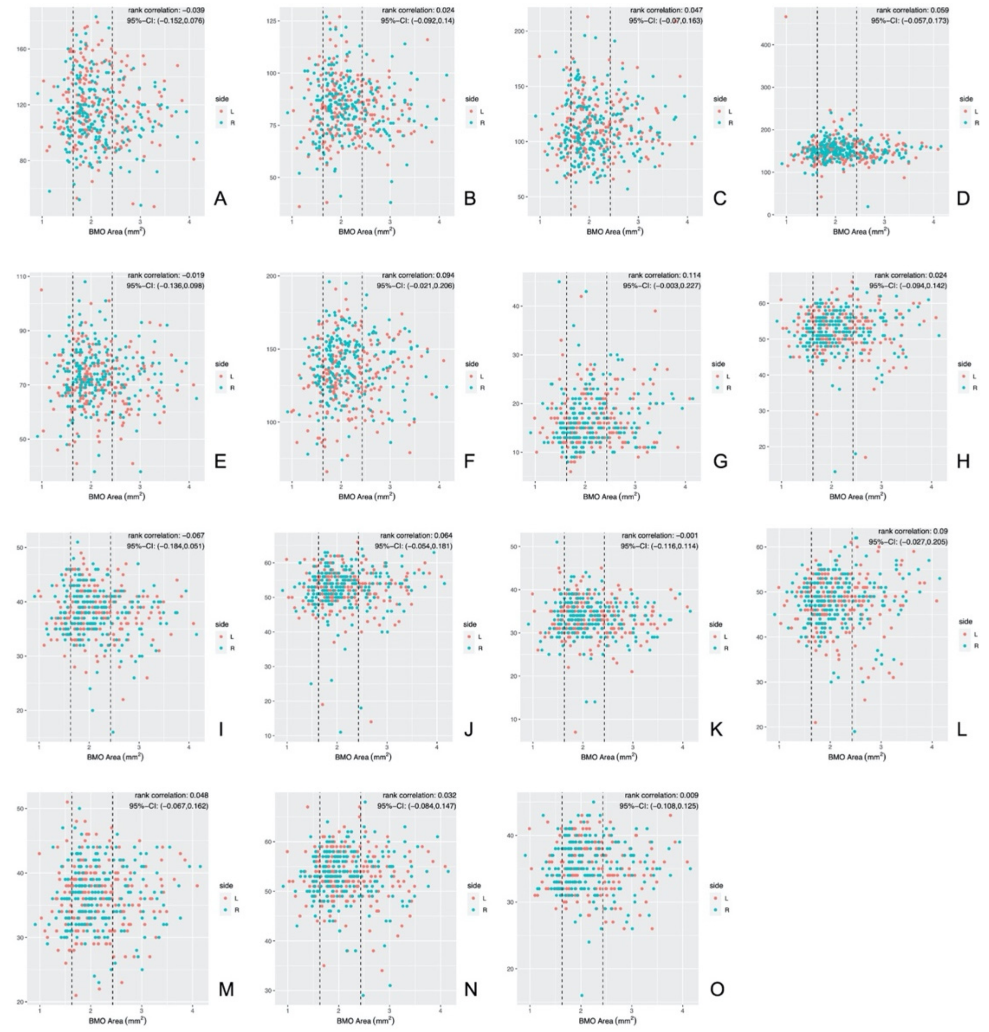

Figure S1. Scatter plots of RNFL sectors in relation to BMOA. Dashed lines show bounds grouping by HRT definition.

L = left eye; R = right eye; A = nasal superior (NS); B = nasal (N); C = nasal inferior (NI); D = temporal inferior (TI); E = temporal (T); F = temporal superior (TS); G = Central area (C); H = Inner nasal (IN); I = Outer nasal (ON); J = Inner inferior (II); K = Outer inferior (OI); L = Inner temporal (IT); M = Outer temporal (OT); N = Inner superior (IS); O = Outer superior (OS).
