# Supplementary material for: Evaluation of Retinal Nerve Fiber Layer and Macular Ganglion Cell Layer Thickness in Relation to Optic Disc Size
Source: J Clin Med. 2023 Mar 24;12(7):2471. doi: 10.3390/jcm12072471 (PMC10095471; doi:10.3390/jcm12072471)
Supplement: Supplementary file 1 [file jcm-12-02471-s001.zip › Table S1.pdf]

Table S1. Differences in individual RNFL and mGCLT sectors between optic disc cohorts based on HRT division. Variable-wise Bonferoni-corrected p values ≤ .05 are highlighted in bold. Median thickness values for groups 1, 2, 3 are given in µm.

RNFL	*Small vs medium*	*Medium vs large*	*Small vs large*	*median thickness small*	*median thickness medium*	*median thickness large*
NS	0.62	0.30	1.00	108.00	115.00	112.00
N	0.11	**0.05**	1.00	80.50	85.00	82.00
NI	0.42	1.00	0.43	103.50	109.00	112.00
TI	**0.03**	0.63	0.48	147.00	153.00	151.00
T	0.75	0.39	1.00	69.50	72.00	71.00
TS	0.06	1.00	0.22	130.50	137.00	138.00
mGCLT						
C	1.00	0.63	0.67	15.00	16.00	16.00
IN	0.46	1.00	1.00	52.50	53.00	53.00
ON	0.06	0.04	1.00	37.00	39.00	38.00
II	**0.04**	1.00	0.42	52.00	54.00	54.00
OI	**0.04**	0.09	1.00	32.00	34.00	33.00
IT	1.00	1.00	1.00	48.50	48.00	49.00
OT	0.05	1.00	0.36	36.00	37.00	37.00
IS	**0.02**	0.35	1.00	52.00	54.00	53.00
OS	**0.01**	0.17	0.80	34.00	35.50	35.00

RNFL sectors: NS = nasal superior; N = nasal; NI = nasal inferior; TI = temporal inferior; T = temporal; TS = temporal superior; mGCLT sectors: C = Central area; IN = Inner nasal; ON = Outer nasal; II = Inner inferior; OI = Outer inferior; IT = Inner temporal; OT = Outer temporal; IS = Inner superior; OS = Outer superior.
