# Supplementary material for: Evaluation of Retinal Nerve Fiber Layer and Macular Ganglion Cell Layer Thickness in Relation to Optic Disc Size
Source: J Clin Med. 2023 Mar 24;12(7):2471. doi: 10.3390/jcm12072471 (PMC10095471; doi:10.3390/jcm12072471)
Supplement: Supplementary file 1 [file jcm-12-02471-s001.zip › Table S2.pdf]

Table S2. Differences in individual RNFL and mGCLT sectors between optic disc cohorts based on quantile division. p values ≤ .05 are highlighted in bold. Median thickness values for the respective groups are given in µm.

RNFL	*Small vs medium*	*Medium vs large*	*Small vs large*	*median thickness small*	*median thickness medium*	*median thickness large*
NS	0.61	1.00	0.76	104.00	113.00	117.50
N	1.00	1.00	1.00	81.00	83.00	80.50
NI	0.58	1.00	0.47	103.00	109.00	114.00
TI	0.45	1.00	0.44	145.00	151.00	158.50
T	1.00	1.00	1.00	73.00	72.00	69.00
TS	**0.04**	1.00	0.66	124.00	136.00	140.00
mGCLT						
C	1.00	0.10	0.33	15.00	15.00	20.00
IN	1.00	1.00	1.00	53.00	53.00	54.50
ON	1.00	1.00	1.00	37.00	38.00	38.50
II	0.38	0.20	0.11	51.00	53.00	55.00
OI	**0.03**	1.00	0.18	31.00	34.00	34.00
IT	1.00	**0.01**	0.07	49.00	48.00	52.00
OT	1.00	0.25	0.37	36.00	37.00	38.50
IS	0.83	1.00	0.82	52.00	53.00	54.00
OS	1.00	1.00	1.00	35.00	35.00	35.50

RNFL sectors: NS = nasal superior; N = nasal; NI = nasal inferior; TI = temporal inferior; T = temporal; TS = temporal superior; mGCLT sectors: C = Central area; IN = Inner nasal; ON = Outer nasal; II = Inner inferior; OI = Outer inferior; IT = Inner temporal; OT = Outer temporal; IS = Inner superior; OS = Outer superior.
